# The Nitrogen-Fixation Island Insertion Site Is Conserved in Diazotrophic *Pseudomonas stutzeri* and *Pseudomonas* sp. Isolated from Distal and Close Geographical Regions

**DOI:** 10.1371/journal.pone.0105837

**Published:** 2014-09-24

**Authors:** Anastasia Venieraki, Maria Dimou, Eleni Vezyri, Alexandros Vamvakas, Pagona-Artemis Katinaki, Iordanis Chatzipavlidis, Anastasia Tampakaki, Panagiotis Katinakis

**Affiliations:** Department of Crop Science, Laboratory of General and Agricultural Microbiology, Agricultural University of Athens, Athens, Greece; University of the West of England, United Kingdom

## Abstract

The presence of nitrogen fixers within the genus *Pseudomonas* has been established and so far most isolated strains are phylogenetically affiliated to *Pseudomonas stutzeri*. A gene ortholog neighborhood analysis of the nitrogen fixation island (NFI) in four diazotrophic *P. stutzeri* strains and *Pseudomonas azotifigens* revealed that all are flanked by genes coding for cobalamin synthase (*cobS*) and glutathione peroxidise (*gshP*). The putative NFIs lack all the features characterizing a mobilizable genomic island. Nevertheless, bioinformatic analysis *P. stutzeri* DSM 4166 NFI demonstrated the presence of short inverted and/or direct repeats within both flanking regions. The other *P. stutzeri* strains carry only one set of repeats. The genetic diversity of eleven diazotrophic *Pseudomonas* isolates was also investigated. Multilocus sequence typing grouped nine isolates along with *P. stutzeri* and two isolates are grouped in a separate clade. A Rep-PCR fingerprinting analysis grouped the eleven isolates into four distinct genotypes. We also provided evidence that the putative NFI in our diazotrophic *Pseudomonas* isolates is flanked by *cobS* and *gshP* genes. Furthermore, we demonstrated that the putative NFI of *Pseudomonas* sp. Gr65 is flanked by inverted repeats identical to those found in *P. stutzeri* DSM 4166 and while the other *P. stutzeri* isolates harbor the repeats located in the intergenic region between *cobS* and glutaredoxin genes as in the case of *P. stutzeri* A1501. Taken together these data suggest that all putative NFIs of diazotrophic *Pseudomonas* isolates are anchored in an intergenic region between *cobS* and *gshP* genes and their flanking regions are designated by distinct repeats patterns. Moreover, the presence of almost identical NFIs in diazotrophic *Pseudomonas* strains isolated from distal geographical locations around the world suggested that this horizontal gene transfer event may have taken place early in the evolution.

## Introduction

The genus *Pseudomonas* includes more than 110 species of agricultural, environmental, biotechnological and clinical importance [1], [2]. The species *Pseudomonas stutzeri* is a non-fluorescent member of the genus *Pseudomonas*. Phylogenetic analysis of 14 sequenced *Pseudomonas* strains belonging to five *Pseudomonas* species, based on 1,705 conserved genes, indicated that *P. stutzeri* A1501 is somewhat distantly related to the other *Pseudomonas* spp. [3]. A phylogenetic tree based on concatenation of four housekeeping genes (16S rRNA, *gyrB*, *rpoB* and *rpoD* genes) from 107 *Pseudomonas* species placed type strain *P. stutzeri* ATCC 17588^T^ in a separate group along with *P. azotifigens*, *P. balearica* and *P. xanthomarina* [2]. Members of *P. stutzeri* were further grouped by DNA-DNA hybridization into at least nineteen genomic groups termed genomovars [4], [5]. *P. stutzeri* occupies diverse ecological niches including marine, soil and sedimentary habitats, clinical specimens and wastewater of chemical industry. Members of the species exhibit metabolic versatility ranging from the utilization of a wide range of organic substrates, degradation of xenobiotics and synthesis of polyhydroxyalkalonates [1].

It was long believed that there were no nitrogen-fixers among strains of the genus *Pseudomonas sensu stricto* [6]. Recently, this belief has been revised, since several nitrogen-fixing strains belonging to *P. stutzeri* have been isolated and characterized from the rhizosphere of gramineous plants like sorghum [7], [8], rice [9], wheat [10] and barley [11]. A diazotrophic *Pseudomonas*, *P. azotifigens*, has also been isolated from a compost pile [12]. Diazotrophic *P. stutzeri* strains have also been isolated from diverse ecological systems such as Galapagos rift near a hydrothermal vent [13] industrial hydrocarbon sludge [14] and wastewater [15].

Genome analysis of the fully sequenced genomes of two diazotrophic *P. stutzeri* A1501 and DSM4166 strains revealed that the genes involved in nitrogen-fixation are clustered in a 49-kb putative nitrogen fixation island [8], [16]. Based on the GC content, the nitrogen-fixation island (NFI) of A1501 strain, consisting of 59 genes, was postulated to be a genomic island acquired through horizontal transfer, and inserted between PST_1301(*cobS*) and PST_1360 (glutathione peroxidise encoding gene, *gshP*) [16]. Draft genome sequences of five diazotrophic *Pseudomonas* strains genomes have become available in the public biological databases: *P. stutzeri* DSM 4166 [8], *P. stutzeri* B1SMN1 [15], *P. stutzeri* KOS6 [14], *P. stutzeri* NF13 [13] and *Pseudomonas azotifigens* DSM 17556^T^ [17].

In addition to nitrogen-fixation ability, the genomes of both diazotrophic *P. stutzeri* strains A1501, DSM4166 and strain *P. stutzeri* ATCC 17588 harbor all the genes required for complete denitrification and nitrate assimilation [8], [16], [18]. Denitrification is a respiratory process by which nitrate is successfully reduced via nitrite, nitric oxide, nitrous oxide and finally to dinitrogen gas by the action of the *narG* or *narA, norB, nirS* and *nosZ* gene products [19]. The vast majority if not all of *P. stutzeri* strains examined so far, harbor the gene (*nosZ*) coding for nitrous oxide reductase which catalyze the final step in denitrification suggesting that denitrification genes comprise part of the core genome of *P. stutzeri* [18], [20]. Nitrate assimilation is a reductive process in which nitrate is first converted to nitrite and then to ammonia by the action of assimilatory nitrate and nitrite reductases encoded by *nasA* and *nasB* genes, respectively [21]. Both genes coding both nitrite- and nitrate reductases are found in members of *Pseudomonas* spp. including diazotrophic *P. stutzeri* [1].

Global transcriptional profiling demonstrated expression of genes coding for enzymes involved in nitrate assimilation, denitrification and nitrite ammonification are also induced that under nitrogen fixing conditions, suggesting that all these processes may be accomplished in parallel [3]. This observation was further supported by the findings that the presence of low concentrations of nitrate [22] or ammonium [23] did not abolish nitrogen fixation ability of *P. stutzeri* A1501 while there is evidence that constitutive expression of *nifA* regulatory gene may result in enhanced nitrogen fixation even under high-ammonia conditions [24]. Recently, it has also been shown that constitutive expression of *nifA* enhanced ammonium excretions by an *amtB1- amtB2* double mutant [25]. Furthermore, integration of a large fragment of *P. stutzeri* A1501 nitrogen fixation island (52 genes corresponding to PST_1302-PST1306 and PST_1313-PST_1359 regions) into a random genome site of *P. fluorescence* Pf-5 converted this bacterium to nitrogen fixer and ammonia producer [26].

The use of molecular approaches has facilitated the development of rapid and simple methods for genetic diversity and genome structure analysis of natural microbial populations [27]. The combined phylogenetic and multilocus DNA sequence analysis of 16S rRNA gene and other sequences (e.g. ITS1 region sequences, housekeeping and functional genes) have been proven reliable tools for comparative genetic diversity of *P. stutzeri* strain [20], [27]. Furthermore, rep-PCR fingerprinting has successfully been used for genetic diversity and relationship of *P. stutzeri* and genome structure analyses [28], [29].

In the present study, bioinformatic analysis demonstrated that the NFI of diazotrophic *P. stutzeri* strains, whose genome have been fully or partially sequenced, is flanked by *cobS* and *gshP* genes. Further analysis demonstrated that the NFI of these diazotrophic *P. stutzeri* strains is also flanked by direct and/or inverted repeats. Subsequently, we examined the phylogenetic affiliation of eleven diazotrophic *Pseudomonas* isolates. Nine *Pseudomonas* isolates grouped along with *P. stutzeri* and two isolates are grouped in a separate clade. Next their genetic diversity was investigated through molecular fingerprinting (Rep-PCR). Finally, we investigated whether these isolates carry a putative NFI which is also flanked by *cobS* and *gshP* genes and analyzed the flanking regions of the NFI for the presence of direct and/or inverted repeats.

## Results

### The nitrogen-fixation island of diazotrophic *P. stutzeri* strains is flanked by cobalamin synthase and glutathione peroxidase genes

**Figure 1:**
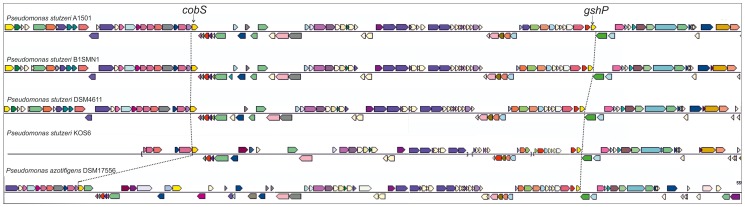
Schematic representation and comparison of the Nitrogen Fixation Islands and flanking genes of diazotrophic *P. stutzeri* strains A1501, DSM4166, B1SMN1, KOS6 and *P. azotifigens* DSM 17556. The nitrogen fixation island of *P. stutzeri* KOS6 was assembled downloading from the Integrated Microbial Genomes (IMG) (https://img.jgi.doe.gov/cgi-bin/w/main.cgi) three contigs (AMCZ01000041, AMCZ01000045 and AMCZ000005) indicated by brackets. The nitrogen fixation island of *P. stutzeri* strains B1SMN1 and *P. azotifigens* DSM 17556 were found in one contig. The colored of arrows are indicating different functional genes as described by IMG.

*P. stutzeri* A1501 harbor a NFI which was postulated to be a genomic island (GI) acquired by horizontal transfer and inserted between *cobS* and *gspH* [16]. Gene neighborhood analysis (http://img.jgi.doe.gov/cgi-bin/w/main.cgi) revealed that the putative NFI of *P. stutzeri* A1501 is also present in the sequenced genomes of diazotrophic *P. stutzeri* strains B1SMN1, KOS6 and DSM4166 and is flanked by *cobS* on one side and a gene homologous to glutathione peroxidase (henceforth referred as *gshP*) on the other (Figure 1). The gene content and gene arrangement of the NFI identified in these strains are almost identical to the NFI of *P. stutzeri* A1501 (Figure 1).The diazotrophic strain *P. stutzeri* NF13 carries all the genes identified in *P. stutzeri* A1501 nitrogen fixation island distributed into two possibly contiguous contigs (Figure S1) but their arrangement is quite different as compared to that found in *P. stutzeri* A1501. A large part of *P. stutzeri* NF13 NFI is flanked by *gspH* and a fragment (164 nt) of the intergenic region *cobS*-PST_1302 but not by the *cobS* gene. The rest of *P. stutzeri* NF13 NFI is found in the second contig which also harbour a small fragment of the intergenic region *cobS*-PST_1302 (Figure S1).

A similar analysis demonstrated that diazotrophic *P. azotifigens* DSM 17556 also carries a putative NFI which is also flanked by *cobS* and *gshP* (Figure 1). The size of *P. azotifigens* DSM 17556 NFI is larger (66.7 kb) as compared to those found in *P. stutzeri* A1501 and DSM4166 which are approximately 49 kb. Nevertheless, the *P. azotifigens* DSM 17556 NFI has similar gene content and arrangement to that of *P. stutzeri* A1501 NFI.

In *P. stutzeri* A1501 NFI, the gene arrangement adjacent to *cobS* is composed of five genes (PST_1302, 1303, 1304, 1305 and 1306). A synteny analysis revealed the presence of homologous genes, in a conserved arrangement in all diazotrophic *P. stutzeri* strains with the exception of the gene cluster of *P. stutzeri* KOS6 NFI which is composed of four genes homologous to PST_1303, PST_1304, PST_1305 and PST_1306. The complete set of the five genes is also found in *P. azotifigens* NFI adjacent to *cobS*. The NFI gene cluster (PST_1359, PST_1358 PST_1357, PST_1356, PST_1357) adjacent to *gshP* is conserved in all diazotrophic *P. stutzeri* strains as well as in *P. azotifigens* DSM 17556^T^ (Figure 1).

### The nitrogen fixation island of diazotrophic *P. stutzeri* strains is flanked by direct and/or inverted repeats

A basic characteristic feature of genomic islands is the presence of short inverted repeats at both sides [33], [34]. It was, therefore, of interest to investigate whether the flanking regions of the NFIs of diazotrophic *Pseudomonas* strains carry short inverted repeats. As flanking regions we define the intergenic region between *cobS* and PST_1302 (henceforth IRLeft) genes and PST_1359 and *gshP* (henceforth IRRight) genes. In *P. stutzeri* KOS6 the CDS coding for glutaredoxin (PST_1302) is absent from this gene cluster, therefore we considered the intergenic region between *cobS* and PST_1303 as IRLeft. To this purpose, we retrieved from all the sequenced genomes of diazotrophic *P. stutzeri* strains the nucleotide sequences of IRLeft (File S1) and IRRight (File S2). The IRLeft of *P. stutzeri* NF13 was constructed by sequences found at the very end of the contig AOBS01000009 and sequences found upstream of glutaredoxin (PST_1302) which is located at the very beginning of the contig AOBS010000070 (Figure S1 and File S1).

Pairwise alignment of the nucleotide sequences of the IRLeft region from the different diazotrophic *P. stutzeri* strains revealed that the nucleotide sequences of *P. stutzeri* A1501, B1SMN1 were identical. IRLeft sequences of *P. stutzeri* DSM 4166 and NF13 were almost identical. Extensive nucleotide sequence homology was identified particularly at the 3′-end and 5′-end of IRLeft of *P. stutzeri* A1501 and DSM 4166 (File S1). The IRLeft region of *P. stutzeri* KOS6 showed no nucleotide sequence homology with the other IRLeft regions. The nucleotide sequence of the latter IRLeft region exhibited an unusual pattern of direct repeats which showed some similarities with the PST_1332-PST_1333 intergenic sequences. The 5′-end region of IRLeft sequences of *P. stutzeri* DSM 4166 and *P. azotifigens* DSM 17566^T^ showed extensive nucleotide sequence identity (File S1).

A similar analysis was also carried out for IRRight (File S2). The nucleotide sequence of *P. stutzeri* A1501, B1SMN1, NF13 and DSM 4166 IRRight regions were almost identical while the 5′-end of IRRight regions of *P. stutzeri* DSM 4166 and KOS6 also shared extensive nucleotide sequence homology (File S2). The 5′-end region of IRLeft sequences of *P. stutzeri* DSM 4166 and *P. azotifigens* 17566 showed extensive nucleotide sequence identity (File S2).

**Figure 2:**
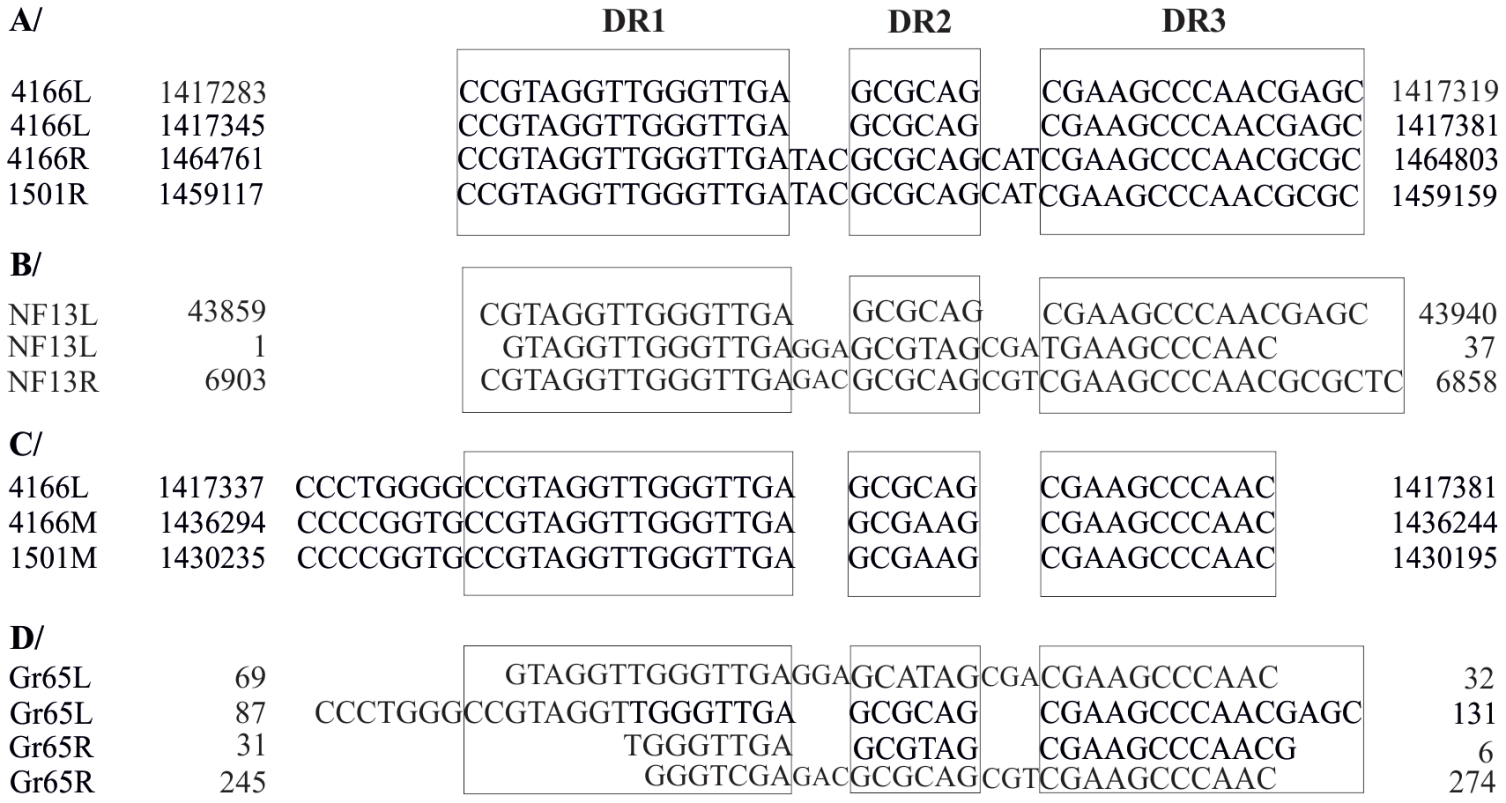
Inverted and/or direct repeats identified in the IRLeft and IRRight regions flanking the nitrogen fixation island of *P. stutzeri* strains and *Pseudomonas* sp. Gr65. The repeats DR1, DR2 and DR3 (boxed) present in the IRLeft and IRRight regions flanking the nitrogen fixation island of *P. stutzeri* A1501 and DSM4166 (A), *P. stutzeri* NF13 (B) and *Pseudomonas* sp. Gr65 (D). The repeats located in the intergenic region between PST_1322- PST_1323 (designated as 1501Μ) and PSTAA_1354- PSTAA_1355 (designated as 4166Μ) (C). The coordinates displayed on the left and the right side of the sequences indicate the position of the sequences in genome of *P. stutzeri* A1501 or DSM4166 (A and C). The coordinates displayed for *Pseudomonas* sp. Gr65 were based on the nucleotide sequences of IRLeft and IRRight found in the files S1 and S2.

Next we examined whether the IRLeft and IRRight carry direct or inverted repeats by employing the OligoRep tool (http://wwwmgs.bionet.nsc.ru/mgs/programs/oligorep/). Analysis of *P. stutzeri* DSM4166 IRLeft and IRRight sequences revealed that both harbor three consecutive short inverted repeats, 14 bp (DR1), 6 bp (DR2) and 11 bp (DR3) (Figure 2). It should also be pointed out that IRLeft carries an extra copy of truncated inverted repeats (Figure 2). This type of inverted repeats was also identified in both IRLeft and IRRight regions of *P. stutzeri* NF13 (Figure 2).The DR1, DR2 and DR3 were also identified in the IRLeft flanking the A1501 strain NFI but not in the IRRight region (Figure 2). A BlastN analysis revealed the presence of DR1, DR2 and DR3 in the intergenic regions of PST_1322 - PST_1323 and PSTAA_1354 - PSTAA_1355, located in the nitrogen fixation island of both A1501 and DSM4166 strains, respectively (Figure 2). Analysis of *P. stutzeri* KOS6 IRLeft and IRRight sequences revealed the presence of a short inverted repeat which however is different that found in *P. stutzeri* DSM4166 (File S3). *P. azotifigens* DSM 17556 IRLeft and IRRight sequences also carry three short direct repeats different from those in the aforementioned diazotrphic *Pseudomonas* strains (File S4).

### Genetic diversity of diazotrophic *Pseudomonas* isolated from the rhizosphere of cereals grown in Greece

**Figure 3:**
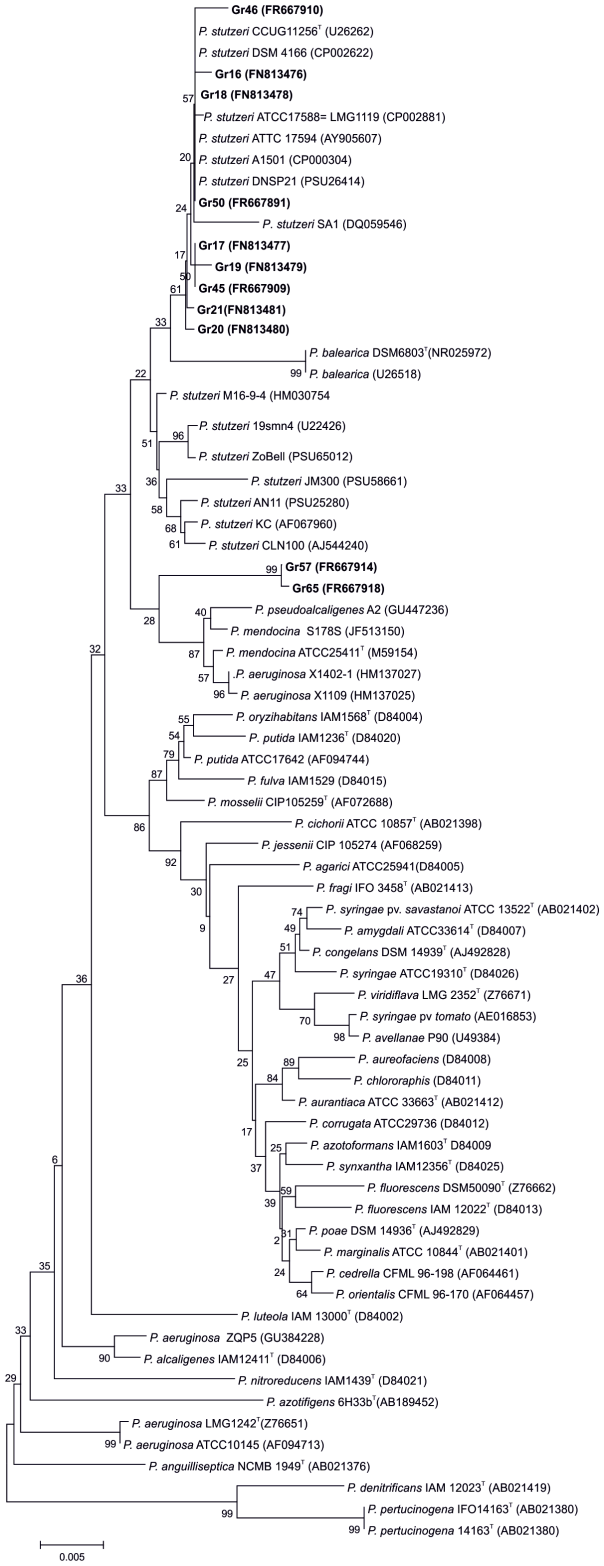
16S rRNA phylogenetic tree. Neighbor-Joining phylogenetic tree of 16S rRNA gene constructed using the partial nucleotide sequence from the 11 *P. stutzeri* isolates and related sequences obtained from NCBI [40]. Numbers shown at nodes indicate bootstrap values (percentage of 1000 replicates). The trees were constructed by the neighbour-joining method using MEGA v.5. Reference strains are highlighted in bold. The bar scale indicates the rates of substitution per nucleotide position. Sequence accession numbers are given in parentheses. ^T^ = type strain.

A collection of eight isolates, seven retrieved from the rhizosphere of wheat and one retrieved from the rhizosphere of barley were previously identified as *P. stutzeri* based on their 16S rRNA gene sequences [10]. We also retrieved two new nitrogen fixing *Pseudomonas* isolates (Gr57 and Gr65) from the rhizosphere of a local barley cultivar and one (Gr46) from the rhizosphere of wheat (Table S1). All (eleven) isolates are nitrogen fixers under microaerophilic conditions as was judged by acetylene reduction assay (Table S1). Pair-wise comparison of the 16S rRNA revealed that most of the strains (Gr16, Gr17, Gr18, Gr19, Gr20, Gr21, Gr45, Gr46 and Gr50) shared homology to *P. stutzeri* strains exceeding 99.5% while both isolates Gr57 and Gr65 exhibited sequence similarities ranging from 97% to 97.2%, respectively when compared to type strains *P. stutzeri* and diazotrophic reference *P. stutzeri* A1501 and DSM4166 strains. Dissimilarity of the 16S rRNA genes more than 1.3% is a strong indication that the isolates under consideration may belong to two groups of different *Pseudomonas* species [11]. To confirm these indices, a 16S rRNA gene phylogenetic tree was constructed (Figure 3). The data indicated that our isolates, with the exception of Gr57 and Gr65, are very closely related to the type strain *P. stutzeri* CCUG1126^T^ and reference diazotrophic strains *P. stutzeri* A1501 and DSM 4166. The isolates Gr57 and Gr65 are placed in a separate branch and hence are referred as *Pseudomonas* sp.Gr65 and *Pseudomonas* sp. Gr65, respectively (Figure 3).

PCR amplification using primers designed from the flanking terminal sequences of the 16S and 23S rRNA genes was performed with chromosomal DNA extracted from the eleven diazotrophic *Pseudomonas* isolates. The size determination of PCR-derived ITS1 products revealed the presence of two distinct products (one small, designated as ITS1S and one larger amplicon designated as ITS1L) with different intensities (the ITS1S being more intense than ITS1L) in the *P. stutzeri* A15 and *P. stutzeri* Gr50 while the PCR-amplified ITS1 products of the *P. stutzeri* DSM4166 and isolates Gr16, Gr17, Gr18, Gr19, Gr20, Gr21, Gr45 and Gr46 were represented by a single band (ITS1S) with a size virtually identical to ITS1S of *P. stutzeri* A15. The PCR-amplicon of strains Gr57 and Gr65 was also represented by a single band with a size slightly larger than ITS1L (data not shown).

Analyses of the nucleotide sequences of all ITS1 regions indicated that all contained two deduced tRNA genes, tRNA^Ala^ and tRNA^Ile^ irrespective of the size and nucleotide differences. *P. stutzeri* A1501 ITS1S nucleotide sequences is virtually identical to the ITS1 sequences from strains DSM4166, ATCC17588, Gr16, Gr17, Gr18, Gr19, Gr20, Gr21, Gr45, Gr46 and Gr50. The ITS1 phylogenenic tree indicated that all isolates clustered with the *P. stutzeri*. (File S5.A).

The *nifD* phylogenetic tree showed that isolates Gr16, Gr17, Gr18, Gr19, Gr20, Gr21, Gr45, Gr46, Gr50 clustered with the diazotrophic strain A1501 and while the strains Gr57 and Gr65 formed a separate clade (File S5.B). Phylogenetic trees constructed using partial sequences of *nirS* (File S5.C) and *napA* (File S5.D) clearly showed that strains Gr57 and Gr65 conformed a separate branch and were adjacent to the defined species such as *Pseudomonas brassicacearum* subsp. *brassicacearum* NFM421 and *P. aeruginosa*, while the phylogenetic position of the other nine strains were closely clustered to *P. stutzeri* DSM4166, ATCC17588^T^ and A1501. On the contrary, the phylogenies of *nosZ* and *narJ* gene showed that all the isolates clustered with *P. stutzeri* (File S5.E and F). The phylogenetic tree constructed using *nasA* (File S5.G) is congruent with that observed for 16S rRNA gene; strains Gr57 and Gr65 formed a separate clade while the other nine strains clustered with *P. stutzeri*. On the contrary, the *nasB* phylogenetic tree revealed that all tested strains were clustered with *P. stutzeri* (File S5.H).

**Figure 4:**
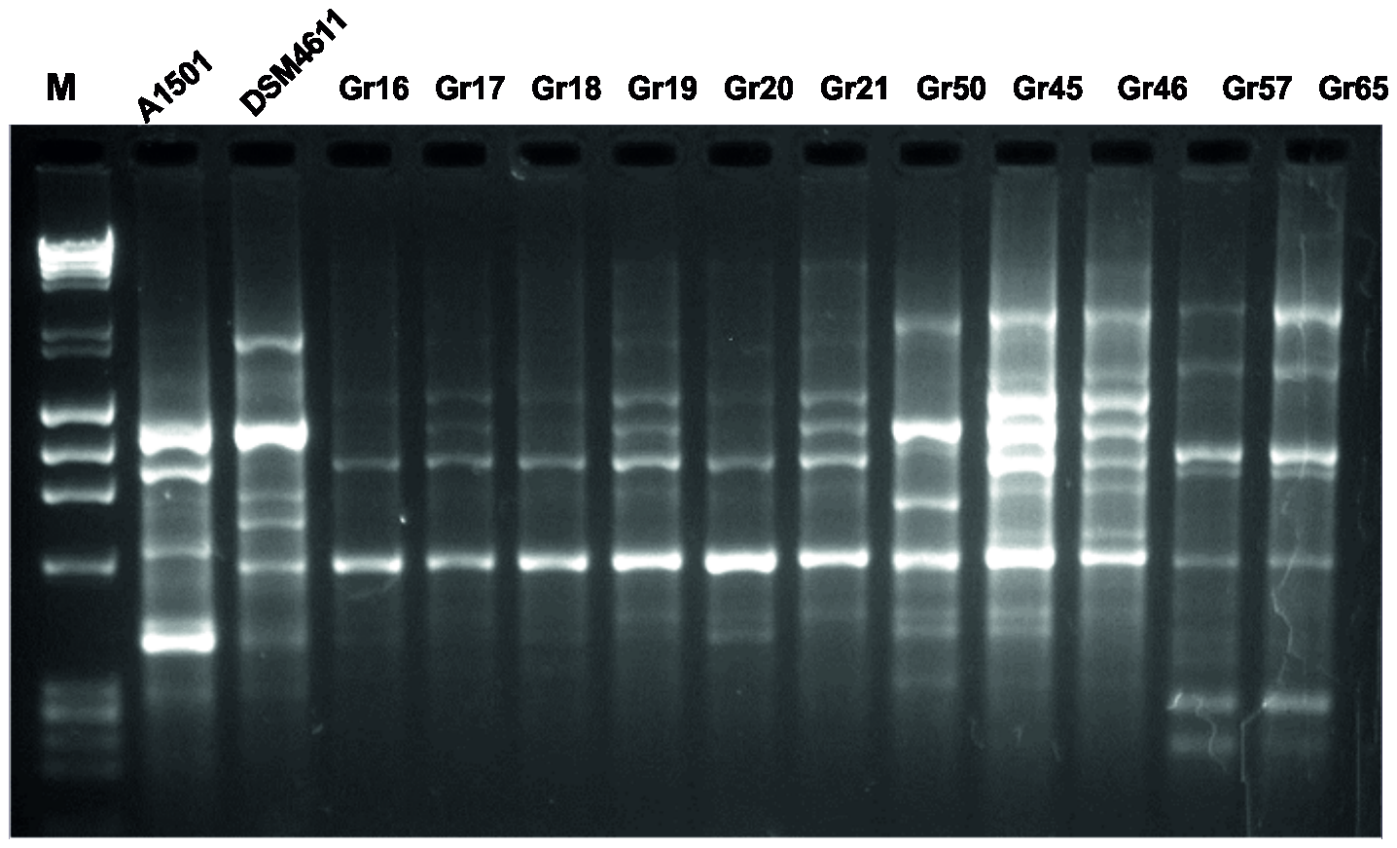
Rep-PCR genomic fingerprinting of *P. stutzeri* strains. Rep-PCR genomic fingerprinting of *P. stutzeri* A1501, *P. stutzeri* DSM4611 and 11 isolates (Gr16, Gr17, Gr18, Gr19, Gr20, Gr50, Gr45, Gr46, Gr57, Gr65). M: DNA ladder λ DNA HindIII and φX174 DNA HaeIII.

To further differentiate the closely related isolates (Gr16, Gr17, Gr18, Gr19, Gr20, Gr21, Gr45, Gr46, Gr50) as well as isolates Gr57 and Gr65, genomic fingerprinting was performed on the eleven strains and two reference strains (A15, DSM4166). The Rep-PCR approach has been proven to generate PCR fingerprints unique to each isolate in *P. stutzeri* and group them at the strain level [28]. Banding profiles generated by Rep primers (Table S2) revealed relatively high diversity among the eleven isolates and classified the isolates into six distinct genotypes, including genotypes from reference strains (Figure 4). The reference strains A15 and DSM4166 exhibited distinct banding patterns, thus representing two distinct genotypes. The banding patterns of the eleven isolates are different from that seen in the reference strains and allowed the grouping into four distinct genotypes: a) isolates Gr45 and Gr46 isolated from the rhizosphere of *T. aestivum* grown at Thessaloniki (Northern Greece) share almost identical banding patterns and may represent one strain (henceforth referred to as Gr45), b) isolates Gr57 and Gr65 isolated from the rhizosphere of *Hordeum vulgare* grown at Larisa (mainland) also exhibited almost identical banding patterns may also represent one strain (henceforth referred to as Gr65), c) isolates Gr16, Gr17, Gr18, Gr19, Gr20 and Gr21 isolated from the rhizosphere of *T. turgidum* var *durum* at Biotia (Eastern mainland) exhibited extensive similarities at the banding patterns may also considered as representing one strain (henceforth referred to as Gr65) and d) isolate Gr50 collected from the rhizosphere of *T. aestivum* at Thessaloniki, exhibited a distinct banding pattern.

### Conservation of the nitrogen fixation island insertion site in our collection of diazotrophic *Pseudomonas* strains

Since the putative NFI of diazotrophic *P. stutzeri* strains whose genomes have been fully sequenced are flanked by genes coding for *cobS* and *gshP* (Figure 1), we asked whether a similar situation may also occur in our collection of diazotrophic *Pseudomonas* strains (Gr19, Gr45, Gr50 and Gr65). To this purpose, two sets of primers were developed based on the nucleotide sequences of *cobS* - PST_1301 and PST_1359 - *gshP* genes, respectively. The amplicons derived following PCR reactions are expected to carry the IRLeft or IRRight intergenic region between *cobS* - PST_1301 and PST_1359 - *gshP* genes. To further establish that our diazotrophic *Pseudomonas* strains carry a NFI two new set of primers were developed based on *cobS*-PST_1307 and PST_1355-*gshP*. These PCR-fragments were cloned, sequenced and the IRLeft and IRRight were determined. Sequences of IRLeft and IRRight are given in Supporting Information (Files S1–S4).

Alignment of the nucleotide sequences of the IRLeft revealed that nucleotide sequences of *Pseudomonas* sp. strains Gr65 and *P. stutzeri* DSM 4166 were identical while IRLeft nucleotide sequences of *P. stutzeri* strains Gr19, Gr45 and Gr50 were identical to the IRLeft sequences of *P. stutzeri* A1501 (File S1). The nucleotide sequences of the IRRight of strains Gr19, Gr45, Gr50, DSM4166 and A1501 were identical while the nucleotide sequence IRRight of strain Gr65 was quite divergent showing extensive similarities to the IRRight of the other strains at the 3-end region (File S2).

Analysis of *Pseudomonas spp.* Gr65 strain IRLeft and IRRight sequences revealed that both harbor the three inverted repeats, DR1, DR2 and DR3, identified in *P. stutzeri* strain DSM4166 (Figure 2). The DR1, DR2 and DR3 were also identified in the *P. stutzeri* Gr19, Gr45 and Gr50 IRRight flanking the nitrogen fixation island found in strain A1501 but not in the IRLeft region (Figure 2).

## Materials and Methods

### Bacterial strains

Bacterial strains used in this study are listed in (Table S1). Reference strains, *Pseudomonas stutzeri* A15 was obtained from the Belgian Coordinated Collections of Micro-organisms, Laboratory for Microbiology of the Faculty of Sciences of Ghent University (BCCM/LMG) and *Pseudomonas stutzeri* CMT.9A = DSM4166 was obtained from German Collection and Cell Culture (DSMZ). *P. stutzeri* strain A1501 is considered a reisolation of strain A15 after a field experiment [24]. In our studies we used *P. stutzeri* A15 and this strain was considered as identical to *P. stutzeri* A1501. All strains were cultured in NA (per liter: peptone 5.0 g, beef extract/yeast extract 3.0 g) medium at 30°C.

### Acetylene reduction assay

The acetylene reduction assay (ARA) was performed on free-living cultures of *Pseudomonas* sp. Gr57, Gr65 and *P. stutzeri* Gr46 isolates as previously described [10]. For the ARA quantification, *P. stutzeri* A15 was used as control.

### DNA extraction, PCR amplification, cloning and phylogenetic analyses

Genomic DNA from 3 ml bacterial cultures of the isolates was extracted using the GenElute Bacterial Genomic DNA kit according to the manufacturer's instructions (Sigma-Aldrich, USA). The quality and quantity of genomic DNA was assessed using a spectrophotometer (Nanodrop ND-1000). The genes encoding 16S rRNA, *nasA, nirS, nirJ, nosZ, nasB*, *nifD* and ITS1 region were amplified using appropriate primers. The primers for amplification together with PCR cycling conditions used are listed in Table S2.The PCR products were separated by electrophoresis in 1.5% (w/v) agarose gel (Invitrogen, UK); the band was excised and purified using a QIAquick Gel Extraction kit (QIAGEN, Germany). The recovered DNA was cloned into the pGEM-T Easy Vector (Promega, USA) according to the manufacturer's protocol. Plasmids containing the appropriate inserts were isolated from 3 ml of bacterial cultures using the QIAprep Spin Miniprep kit, according to the manufacturer's instructions (QIAGEN). Purified plasmids were commercially sequenced (Eurofin MWG, Germany) in both directions. Phylogenetic trees based on nucleotide sequences of the 16S rRNA, *nasA, nirS, nirJ, nosZ, nasB*, *nifD* gene and ITS1 region fragments were constructed with Molecular Evolutionary Genetics Analysis software version 5.0 using the neighbor-joining algorithm (1,000 bootstrap replication) [30].

### Nucleotide Sequence Accession Numbers

The nucleotide sequence data have been submitted to the GenBank database under accession numbers FN813476 to FN813481, FR667909, FR667910, FR667891, FR667914, FR667918 (16S rRNA sequences), FR732002-FR732007, FR732012, FR732013, FR870227, HE813987-HE813989 (ITS1 clones), FR728627 to FR728633, HE813991 and HE813992 (*nifD* clones), HE814015 to HE814025 (*nirS* clones), HE814004 to HE814014 (*napA* clones), HE814026 to HE814036 (*nosZ* clones), HE813993 to HE84003 (*narJ* clones), HE814048 to HE814058 (*nasA* clones) and HE814037 to HE814047 (*nasB* clones), HF951693-HF951703 (glutathione peroxidise-ferredoxin IGS), HF951704-HF951714 (cobalamin synthase- glutaredoxin IGS).

## Discussion

The diazotrophic isolates described in the present study as well as the reference strains are representatives of culturable *P. stutzeri* strains isolated from the rhizosphere of various gramineous plant species (wheat, barley, rice and sorghum) grown in distal geographical locations (China, Germany and Greece) or in close geographical location (Greece). Based on phylogenetic analysis of the 16S rRNA, nine isolates obtained from the rhizosphere soils of wheat clustered with type strains of *P. stutzeri* such as *P. stutzeri* ATCC17588, while isolates Gr57 and Gr65 collected from the rhizopshere of barley are grouped to an adjacent clade. These indices are also supported by the phylogenetic trees obtained from the concatenation of *napA*, *nirS*, *nasB* and *nifD* genes.

It has been reported that *P. stutzeri* isolates with similar Rep-PCR banding patterns also exhibited high DNA sequence homology as inferred by DNA-DNA hybridization analysis [28]. Thus, it could be argued, based on the distinct Rep-PCR banding patterns, that there is a genetic heterogeneity among diazotrophic *P. stutzeri* strains Gr19, Gr45 and Gr50 isolated from the rhizosphere of *T. aestivum*, *T. durum* and *H. vulgare* respectively grown in relatively close geographical regions (less than 300 km distance from each other) in Greece. In general, the plant cultivar and/or plant species is assumed to have a major effect on the selection of microorganisms colonizing the rhizosphere [31]. The colonization of different plant species and/or plant cultivars by different diazotrophic *P. stutzeri* genospecies may be attributed to different environmental factors and soil characteristics of the sampling sites and/or to selection imposed by the plant cultivar and/or plant species, as reported for other diazotrophic soil bacteria such as *Sinorhizobium meliloti* and *S. medicae* [32].

Genomic islands (GIs) are large (5-600 kb) chromosomal regions mostly detected in the vicinity of stable RNA genes (tRNA, tmRNA) and typically flanked by direct and/or inverted repeats [33]. They are acquired by horizontal gene transfer and usually confer traits that increase fitness, adaptation to specific habitats, metabolic proficiency or virulence [34]. GIs can be excised to form circular intermediates and are defined as mobile GIs. The mobile GIs typically contain mobility elements (like integrase, transposase or recombinase genes) which catalyze the GIs excision and/or insertion, although in some cases these gene(s) might have been lost resulting in “anchored genomic islands” [33]. The GIs frequently recognized insertion ‘hotspots’, such as tRNA/tmRNA gene and small non-coding RNA gene [35], [36]. Recently the 3′-end of guanosine monophosphate synthetase gene (*guaA*) has been reported as insertion site of GIs in a number of sequenced microbial genomes [37].

The *P. stutzeri* A1501 genome, based on abnormal GC content, appears to harbor a putative NFI [16]. None of the NFIs identified in diazotrophic *P. stutzeri* strains A1501, KOS6, NF13, B1SMN1, DSM4166 and *P. azotifigens* DSM17556 contain mobile genetic elements, such as integrase and transposase genes, suggesting that these GIs are not self-mobilizable [34], [38], [39]. On the other hand, bioinformatic analysis demonstrated that the genes comprising the putative NFI are contiguous and flanked by *cobS* genes on one side and *gspH* on the other side in all diazotrophic *P. stutzeri* strains (A1501, KOS6, B1SMN1 and DSM4166) and *P. azotifigens* DSM17556^T^, with the exception of *P. stutzeri* NF13.

Our data also provided evidence that our collection of diazotrophic *P. stutzeri* and *Pseudomonas* sp. Gr65 strains harbor a putative NFI which is located between the genes coding for *cobS* and *gshP*, although the complete organization and gene arrangement of these NFIs is not known. However, we provided evidence that the gene organization of the regions located upstream and downstream of *cobS* and *gshP* genes respectively are conserved. Taken together, our data suggest that in *Pseudomonas* the region between *cobS* and *gshP* genes may be a hot spot for insertion of NFI. These indices are further corroborated by neighbourhood region analyses (http://img.jgi.doe.gov/cgi-bin/w/main.cgi) which revealed that *cobS* and *gshP* genes are closely arranged in most of *P. stutzeri* strains (not fixing nitrogen) and the majority of *Pseudomonas* species the genes coding for. Thus, it will be of interest to investigate whether sites between *cobS* and *gshP* genes are convenient for the construction *Pseudomonas* recombinant nitrogen-fixing bacteria.

The putative nitrogen fixation island identified in the sequenced genome of *P. stutzeri* DSM 4166, although does not harbor genes involved in motility and is not associated to a tRNA gene, is flanked by inverted repeats located in the intergenic regions between of *cobS*-glutaredoxin (PSTAA_1334) as well as between glutathione peroxidase-flavodoxin (PSTAA_1391) encoding genes. Interestingly, these inverted repeats (DR1, DR2 and DR3) were also identified in the flanking region of *Pseudomonas* sp. Gr65 and in *P. stutzeri* NF13. A different type of inverted repeats was also identified in the flanking region of *P. stutzeri* KOS6. On the other hand, our bioinformatics analysis and experimental data indicated that *P. stutzeri* A1501, B1SMN1, Gr19, Gr45 and Gr50 strains harbour only one set of DR1, DR2 and DR3 repeats located in the IRRight region. The presence of inverted direct repeats either on one side or on both sides of NFIs might be reminiscent of a motility apparatus which might have been lost through a series of recombination events rendering the NFI immobilizable. Anchored genomic islands have been identified in a number of bacteria [35] including the uropathogenic *E. coli* CFT073 [39] and *Pseudomonas aeruginosa* clone C [39]. The absence of the whole set of direct repeats from IRLeft in diazotrophic *P. stutzeri* strains A1501, G19, Gr45, Gr50 and B1SMN1 raises the question whether an unknown evolutionary adaptation process, at transitory state is taking place, leading to a permanent anchoring of the nitrogen-fixation island on the chromosome.

Our data indicated that all the diazotrophic bacteria harboring the above described nitrogen-fixation island are members of the species *Pseudomonas*. Thus the finding that the highly conserved NFI is present in *Pseudomonas* strains isolated from various locations around the world suggests that these genes were obtained early in the evolution of this species.The observation that a nearly identical copy of the whole set of the direct repeats is present in the intergenic region between the genes PST_1323-PST_1324 and PSTAA_1354-PSTAA_1355 located in the middle of the NFI island of both diazotrophic strains combined with the non-contiguous organization of *P. stutzeri* NF13 NFI suggest that the formation of the contiguous NFI may be the result of at least two recombination events.

In conclusion we have presented data supporting the view that all diazotrophic *P. stutzeri* strains A1501, DSM4166, Gr19, Gr45, Gr50, KOS6 and B1SMN1, *P. azotifigens* DSM 17556 and *Pseudomonas* spp. strain Gr65 isolated from distal geographical locations such as China, Germany, Spain, Japan, Russia and Greece harbor a putative NFI which is located between the *cobS* and *gshP* genes rendering this region as hot spot for insertion of NFIs. The absence of inverted repeats in NFI flanking regions in some diazotrophic *P. stutzeri* strains highlights the possible presence of an unknown mechanism rendering NFI anchored. It is obvious that immobilization of the NFI confers competitiveness in the acquired *Pseudomonas* strains because enhances their metabolic capacities. Recombinant nitrogen fixing *Pseudomonas* have been created by the integration of the *P. stutzeri* NFI into a random position of their genomes [26]. The inserted NIF appears to stably incorporate into their genome enabling the recombinant strains to fix nitrogen in the presence or absence of ammonium [26]. Since *P. stutzeri* strains are amenable to natural transformation [1] it would be of interest to investigate whether exposure of non-nitrogen fixing *P. stutzeri* strains to genomic DNA carrying the NFI might convert them to nitrogen fixers.

## Supporting Information

Figure S1 Schematic presentation of *P. stutzeri* NF13 and *P. stutzeri* A1501 nitrogen fixation island gene clusters. The *P. stutzeri* A1501 nitrogen fixation island (middle). The *P. stutzeri* NF13 contig AOBS010000070 (upper) harbors the gene clusters PST_1302-1306, PST_1308-1309, PST_1313-1323 and PST_1349-1359 and is flanked by a fragment (164 nt) of the intergenic region *cobS*-PST_1302 (IRLeft) sequences and *gshP*. The *P. stutzeri* NF13 contig AOBS01000009 (low) harbors the gene cluster PST_1324-PST_1348 and is flanked by small fragment (59 nt) of the intergenic region IRLeft sequences (cobS-intergenic).The *cobS* gene is located at the 3′end of contig AOBS01000009. The whole nucleotide sequence of *P. stutzeri* NF13 IRLeft is shown in file S1.
(TIF)

File S1 List of IRLeft Sequences and Pairwise alignment of different *P. stutzeri* IRLeft sequences.
(DOCX)

File S2 List of IRRight Sequences and Pairwise alignment of different *P. stutzeri* IRRight sequences.
(DOCX)

File S3 Nucleotide sequences of *P. stutzeri* KOS6 IRLeft and IRRight.
(DOCX)

File S4 Nucleotide sequences of *P. azotifigens* DSM 17556 IRLeft and IRRight.
(DOCX)

File S5 Phylogenetic trees based on ITS1 (A), *nifD* (B), *narJ* (C), *napA* (D), *nirS* (E), *nosZ* (F), *nasA* (G) and *nasB* (H) showing the relationships among the *Pseudomonas* strains and references strains.
(DOCX)

Table S1 Beneficial properties of *Pseudomonas* strains used in this study.
(DOCX)

Table S2 List of primers used and PCR cycling conditions.
(DOCX)
