# Supplementary material for: The Nitrogen-Fixation Island Insertion Site Is Conserved in Diazotrophic Pseudomonas stutzeri and Pseudomonas sp. Isolated from Distal and Close Geographical Regions
Source: PLoS One. 2014 Sep 24;9(9):e105837. doi: 10.1371/journal.pone.0105837 (PMC4174501; doi:10.1371/journal.pone.0105837)
Supplement: File S1 — List of IRLeft Sequences and Pairwise alignment of different P. stutzeri IRLeft sequences. (DOCX) [file pone.0105837.s002.docx]

**File S1. List of Sequences of IRLeft**

List of IRLeft sequences extracted either from the sequenced genomes of *P. stutzeri* A1501 (1501L), *P. stutzeri* M1SMN1 (M1SMN1L), *P. stutzeri* DSM 4166 (4166L), *P. stutzeri* NF13 (NF13L), *P. stutzeri* KOS6 (KOS6L), *P. azotifigens* DMS 17556 (17556L) or from the sequenced intergenic region between *cobS* - PST_1301 of *P. stutzeri* Gr19 (Gr19L), *P. stutzeri* Gr45 (Gr45L) *P. stutzeri* Gr50 (Gr50L) and *Pseudomonas sp.* Gr65 (Gr65L). Nucleotide sequences of 1501L, M1SMN1L, Gr19L. Gr45L and Gr50L are identical. The *P. stutzeri* NF13 IRLeft nucleotide sequences was constructed by joining the nucleotide sequences located at the 5’end of contig AOBS01000009 (59 nt) and the 5’end nucleotide sequences of contig AOBS010000070(164 nt).

>1501L

GGTTAGGTTGGCCTGAATTCGGTGTGTATCCCCCGGAGATCAGCTTCGCCTCGGCACGCTCAGCCTGCACTCGCCCCAGCC

>M1SMN1L

GGTTAGGTTGGCCTGAATTCGGTGTGTATCCCCCGGAGATCAGCTTCGCCTCGGCACGCTCAGCCTGCACTCGCCCCAGCC

>4166L

GGTTGGGCTGGCCTTGAATTCGGTGTGTCATGTTGGGCTTCGTCGCTATGCTCCTCAACCCAACCTACCAGGCTGCGCGATCCAACCCTGGGGCCGTAGGTTGGGTTGAGCGCAGCGAAGCCCAACGAGCGAGGTATCAGGCCACGAATTCGTCACGCCATCGGTCTTTTCAACGCCCCGGAGATCATCTTCGCCTCGGCACGTTCAGCCTGCGCTCGCTCCCGCC

>NF13L

NNNNNNNGGCTGGGTTGAGCGCAAGAGCCTGTTGGGCTTCATCGCTACGCTCCTCAACCCAACCTACNNNNNNNNNNNNNNNNNNNNAGGGTCGTAGGTTGGGTTGAGCGCAGCGAAGCCCAACGAGCGCGGTGTCAGGCCACGGATTCGTCACGCCATCGGTCTTTTCGAAGCCCCGAAGACCATCTTCGCCTCGGCACGCTCAGCCTATGCTCGCCCCACCTTAGGTTTCCGGCGCGAGGGCGGCATCG

>KOS6L

GTTGTGGAGCAGGCCCCCGCCTGCACAGCGGAAATGCATGCTCCAGACCCAAAAGCATCGCCCCGAGGTCGGGCCTCCCACAAAGGCAGCGAGTGCACACCATGCCCGGTGGGAGGCGCGCCCTCGCGGCGAAGCAGGCTGAGGCCTGCCGGAAACTCACCGGCAAGCCATCGATCTTTCCGGTGCCGCCATGCATCTGGTCGCGGCCTGACATTAGGCTGCTATTGCGCAGCGGAAATGCACGCTCCAGACCCAAAAGCATCGCCCCGAGGTCGGGCCTCCCGCAAAGGCAGCGAGCGCACACAGGCCTCGTGGGAGGCGCGACCTCGCGGCGAAGCAGGCTAAGGCCTGCCGGAAGCTCACCGGCAGCCATCGATCTTTCCGGTGCCGCCATCTGGTCGCGGCCTGACATTAGGCTGCTATTGCGCAGCGGGAATGCACGCTCCAGGCCCAAAAGCATGGCCCCGAGGTCGGGCCTCCCGCAAAGGCAGCGAGCGCACACAGGCCTCGTCGTGGGAGGCGCGCCCTCGCGGCGAAGCAGGCCGCAGGCCTGCCGGAAGAATCTGCATGCGC

>17556L

CGTGACGTGAGCCGGCGGTTGCTGCCGCTCTGGCTGGCCGCGGGTGCGTCGATGCTGGCCCTGGCCTGGTGGGGCTGGGCGCAGGGCGGGATGGCGCTGCTGCAGCTGGGCGTCGGAGTCTGCTGAGCGGGGCGCGGGCGTCGGGCTTTTCGTGAGCGGTCTCGACCCGTGAACTCAAGGGGCCAGGTTCGCGGTCGAGACCGCTCCCACAAAAGCATCGCCCCGAGGGCGGGCCTCCCACGAAAGGCGATTCTGTGGCAGGCGCGCCCTCGCGGCGATGCAGGCCGCAGGCCTGCCAGAATCTCATCGGTCTTTCAACGCCCCGGAGATCATCTTCGCCTCGGTACGATCAGCCTACGCTCGCCCCTG

>Gr65L

GGTTGGGCTGGCCTTGAATTCGGTGTGTCATGTTGGGCTTCGTCGCTATGCTCCTCAACCCAACCTACCAGGCTGCGCGATCCAACCCTGGGGCCGTAGGTTGGGTTGAGCGCAGCGAAGCCCAACGAGCGAGGTATCAGGCCACGAATTCGTCACGCCATCGGTCTTTTCAACGCCCCGGAGATCATCTTCGCCTCGGCACGTTCAGCCTGCGCTCGCTCCCGCC

>Gr19L

GGTTAGGTTGGCCTGAATTCGGTGTGTATCCCCCGGAGATCAGCTTCGCCTCGGCACGCTCAGCCTGCACTCGCCCCAGCC

>Gr45

GGTTAGGTTGGCCTGAATTCGGTGTGTATCCCCCGGAGATCAGCTTCGCCTCGGCACGCTCAGCCTGCACTCGCCCCAGCC

>Gr50L

GGTTAGGTTGGCCTGAATTCGGTGTGTATCCCCCGGAGATCAGCTTCGCCTCGGCACGCTCAGCCTGCACTCGCCCCAGCC

**Pairwise alignment of different *P. stutzeri* IRLeft sequences**

Alignment of *P. stutzeri* A1501 IRLeft (1501L) and *P. stutzeri* DSM 4166 IRLeft (4166L). Asterisks indicate nucleotide identities.

1501L GGTTAGGTTGGCCTG-AATTCGGTGTG

4166L GGTTGGGCTGGCCTTGAATTCGGTGTGTCATGTTGGGCTTCGTCGCTATGCTCCTCAACC

****.** ****** ***********

1501L

4166L CAACCTACCAGGCTGCGCGATCCAACCCTGGGGCCGTAGGTTGGGTTGAGCGCAGCGAAG

1501L TATCCCCCG

4166L CCCAACGAGCGAGGTATCAGGCCACGAATTCGTCACGCCATCGGTCTTTTCAACGCCCCG

* *****

1501L GAGATCAGCTTCGCCTCGGCACGCTCAGCCTGCACTCGCCCCAGCC

4166L GAGATCATCTTCGCCTCGGCACGTTCAGCCTGCGCTCGCTCCCGCC

******* *************** *********.***** **.***

Alignment of *P. stutzeri* NF13 IRLeft (NF13L) and *P. stutzeri* DSM 4166 IRLeft (4166L). Asterisks indicate nucleotide identities.

4166L GGTTGGGCTGGCCTTGAATTCGGTGTGTCATGTTGGGCTTCGTCGCTATGCTCCTCAA

NF13L NNNNNNNGGCTGGGTTG---AGCGCAAGAGCCTGTTGGGCTTCATCGCTACGCTCCTCAA

****** * : ** :. . *.***********.****** *********

4166L CCCAACCTACCAGGCTGCGCGATCCAACCCTGGGGCCGTAGGTTGGGTTGAGCGCAGCGA

NF13L CCCAACCTACNNNNNNNNNNNNNNNNNNNNNAGGGTCGTAGGTTGGGTTGAGCGCAGCGA

********** . .. . . . ..*** ************************

4166L AGCCCAACGAGCGAGGTATCAGGCCACGAATTCGTCACGCCATCGGTCTTTTCAACGCCC

NF13L AGCCCAACGAGCGCGGTGTCAGGCCACGGATTCGTCACGCCATCGGTCTTTTCGAAGCCC

*************.***.**********.************************.*.****

4166L CGGAGATCATCTTCGCCTCGGCACGTTCAGCCTGCGCTCGCTCCCGCC

NF13L CGAAGACCATCTTCGCCTCGGCACGCTCAGCCTATGCTCGCCCCACCTTAGGTTTCCGGC

**.*** ****************** *******. ****** **. *

4166L ---------------

NF13L GCGAGGGCGGCATCG

Alignment of *P. azotifigens* DSM 17556 IRLeft (17556L) and *P. stutzeri* DSM 4166 IRLeft (4166L). Asterisks indicate nucleotide identities.

4166L ------------------------------------------------------------

17556L CGTGACGTGAGCCGGCGGTTGCTGCCGCTCTGGCTGGCCGCGGGTGCGTCGATGCTGGCC

4166L -------GGTTGGGCTGG----------------------------------------CC

17556L CTGGCCTGGTGGGGCTGGGCGCAGGGCGGGATGGCGCTGCTGCAGCTGGGCGTCGGAGTC

*** ** **** *

4166L TTGAATTCGGTGTGTCATGTTGGGCTTCGTCGCTATGCTCCTCAACCCAA----------

17556L TGCTGAGCGGGGCGCGGGCGTCGGGCTTTTCGTGAGCGGTCTCGACCCGTGAACTCAAGG

* *** * :.: *** * * * * ****

4166L -----------CCTACCAGGCTGCGCGATCCAACCCTGGGGCCGTAGGTTGGGTTGAGCG

17556L GGCCAGGTTCGCGGTCGAGACCGCTCCCACAAAAGCATCGCCCCGAGGGCGGGCCTCCCA

** *** *

4166L C---------------AGCGAAGCCCAACGAGCGAGGTATCAGGCCACGAATTCGTCACG

17556L CGAAAGGCGATTCTGTGGCAGGCGCGCCCTCGCGGCGATGCAGGCCGCAGGCCTGCCAGA

* ****** **

4166L CCATCGGT--CTTTTCAACGCCCCGGAGATCATCTTCGCCTCGGCACGTTCAGCCTGCGC

17556L ATCTCATCGGTCTTTCAACGCCCCGGAGATCATCTTCGCCTCGGTACGATCAGCCTACGC

* ******************************************** ***

4166L TCGCTCCCGCC

17556L TCGCCCCTG--

**** ** *
