# Supplementary material for: The Nitrogen-Fixation Island Insertion Site Is Conserved in Diazotrophic Pseudomonas stutzeri and Pseudomonas sp. Isolated from Distal and Close Geographical Regions
Source: PLoS One. 2014 Sep 24;9(9):e105837. doi: 10.1371/journal.pone.0105837 (PMC4174501; doi:10.1371/journal.pone.0105837)
Supplement: File S2 — List of IRRight Sequences and Pairwise alignment of different P. stutzeri IRRight sequences. (DOCX) [file pone.0105837.s003.docx]

**File S2. List of IRRight Sequences**

List of IRRight sequences extracted either from the sequenced genomes of *P. stutzeri* A1501 (1501L), *P. stutzeri* M1SMN1 (M1SMN1R), *P. stutzeri* DSM 4166 (4166R), *P. stutzeri* NF13 (NF13R), *P. stutzeri* KOS6 (KOS6R) or from the sequenced intergenic region between PST_1359 - *gshP* genes of *P. stutzeri* Gr19 (Gr19R), *P. stutzeri* Gr45 (Gr45R) and *P. stutzeri* Gr50 (Gr50R) and *Pseudomonas sp.* Gr65 (Gr65R). The nucleotide sequences of 1501R, 4166R, B1SNM1R, G19R, Gr45R and Gr50R are identical.

>1501R

CCATGCGTTGAGCTTCGCTGCACGCCCGGCCCCGACCTACGCCTCGTAATCCGTAGGTTGGGTTGATACGCGCAGCATCGAAGCCCAACGCGCTCCGAAGCGCAGCTCGGCGGCGATCTGTGCGAACCGTTGCCCGGCGGCCGTGGGCGGAGTAGCGTGATCGCGAACCGAGGAAGGAGATTCGCC

>4166R

CCATGCGTTGAGCTTCGCTGCACGCCCGGCCCCGACCTACGCCTCGTAATCC**GTAGGTTGGGTTGA**TACGCGCAGCATCGAAGCCCAACGCGCTCCGAAGCGCAGCTCGGCGGCGATCTGTGCGAACCGTTGCCCGGCGGCCGTGGGCGGAGTAGCGTGATCGCGAACCGAGGAAGGAGATTCGCC

>M1SNM1R

CCATGCGTTGAGCTTCGCTGCACGCCCGGCCCCGACCTACGCCTCGTAATCCGTA**GGTTGGGTT**GATACGCGCAGCATCGAAGCCCAACGCGCTCCGAAGCGCAGCTCGGCGGCGATCTGTGCGAACCGTTGCCCGGCGGCCGTGGGCGGAGTAGCGTGATCGCGAACCGAGGAAGGAGATTCGCC

>FN13R

ACGCTCGTTGAGCTTCGTTCGGCTGTAGCCACGCGTCGGGCTTCGCTGCGCTCAGGCTGCGTACCCCGGCCCCGACCTACGCCTCGTAATCCGTAGGTTGGGTTGAGACGCGCAGCGTCGAAGCCCAACGCGCTCCGAAGCGCAGCTCGGCGGCGATCTATGCGAACCGTTGCCCGGCGGCCGTGGGCGGAGTAGCGTGATCGCGAACCGAGGAAGGAGATTCGCC

>KOS6R

CCTTGCGTCGGGCTTCGCTGTGCTCAGTGAAGCCCAACTCAAATGAAGTGTTGCCCAACGTTTCACGGACTCGCAGCAGTCGTTGCCCG**GCGCTCGCTG**GCGGAGTAGCGTGACTGCAAACCGAGGACGGAGAATCGAC

>17556R

GCTCGCCGGGCCTTCGCTGCGCCGAATCCTGGCGACCCAGCCCAACCTGCGTTCCCCGGTGTAACCTGGACAAGCTCGCCCCGAGATCGGGCCTCCCACAAAAGCGGCGTCGCCCACCGAGTCCGGTGGGAGGCACCCTCGCGGCGAACGCGGGCGCGTAGAAGCCCAACGTTTTTCCTACGCCGAGGCTCGAGTTCAACAGCGATCCGCCCGAATCGTTGCCCGGCGGGCATGGGCGGAGTAGCGTGGCTGTGAAACCGACAATGGAGATTCGCC

>Gr65R

CCATGCGTTGGGCTTCGCTACGCTCAACCCAGGCGGCCCCGCCGAACCAACGATGTCGGCGGGGATGGTAGGTTGGATCGGACGGCGTTCCGCTGAAACGCGTAGCGTCGAAGCCCAGCTCACACGAAGGTAACGCTTGGCACCCGTCCCGTTGGGGGCTTGGCTTGGTTGTGACCGTGCGTTGGGCTTGGCTGTGCCCAGGCTGCGCGCCCCGACTCAACAGTTGGATGTCGACGGGGTGATCGGGTCGAGACGCGCAGCGTCGAAGCCCAACTCACATGAAGGCGATGCCCAACGTTTCACGGGCTCGCATGAGCCGTTGCCCGGCGACCGTGGGCGGAGTAGCGTGACTGCAAACCGACGATGGAGACTCGCC

>Gr19R

CCATGCGTTGAGCTTCGCTGCACGCCCGGCCCCGACCTACGCCTCGTAATCCGTAGGTTGGGTTGATACGCGCAGCATCGAAGCCCAACGCGCTCCGAAGCGCAGCTCGGCGGCGATCTGTGCGAACCGTTGCCCGGCGGCCGTGGGCGGAGTAGCGTGATCGCGAACCGAGGAAGGAGATTCGCC

>Gr45R

CCATGCGTTGAGCTTCGCTGCACGCCCGGCCCCGACCTACGCCTCGTAATCCGTAGGTTGGGTTGATACGCGCAGCATCGAAGCCCAACGCGCTCCGAAGCGCAGCTCGGCGGCGATCTGTGCGAACCGTTGCCCGGCGGCCGTGGGCGGAGTAGCGTGATCGCGAACCGAGGAAGGAGATTCGCC

>Gr50R

CCATGCGTTGAGCTTCGCTGCACGCCCGGCCCCGACCTACGCCTCGTAATCCGTAGGTTGGGTTGATACGCGCAGCATCGAAGCCCAACGCGCTCCGAAGCGCAGCTCGGCGGCGATCTGTGCGAACCGTTGCCCGGCGGCCGTGGGCGGAGTAGCGTGATCGCGAACCGAGGAAGGAGATTCGCC

**Alignment of *P. stutzeri* IRRight sequences**

Alignment of *P. stutzeri* A1501 IRRight (1501R), *P. stutzeri* FN13 IRRight (FN13R), *P. stutzeri* M1SMN1 IRRight (M1SMN1R)and *P. stutzeri* DMS 4166 IRRight (4166R). Asterisks indicate nucleotide identities.

M1SNM1R ----------------------------CCATGCGTTGAGCTTCGCTGCACG--------

FN13R ACGCTCGTTGAGCTTCGTTCGGCTGTAGCCACGCGTCGGGCTTCGCTGCGCTCAGGCTGC

4166R ----------------------------CCATGCGTTGAGCTTCGCTGCACG--------

1501R ----------------------------CCATGCGTTGAGCTTCGCTGCACG--------

*** **** *.**********.*

M1SNM1R ----CCCGGCCCCGACCTACGCCTCGTAATCCGTAGGTTGGGTTGATACGCGCAGCATCG

FN13R GTACCCCGGCCCCGACCTACGCCTCGTAATCCGTAGGTTGGGTTGAGACGCGCAGCGTCG

4166R ----CCCGGCCCCGACCTACGCCTCGTAATCCGTAGGTTGGGTTGATACGCGCAGCATCG

1501R ----CCCGGCCCCGACCTACGCCTCGTAATCCGTAGGTTGGGTTGATACGCGCAGCATCG

****************************************** *********.***

M1SNM1R AAGCCCAACGCGCTCCGAAGCGCAGCTCGGCGGCGATCTGTGCGAACCGTTGCCCGGCGG

FN13R AAGCCCAACGCGCTCCGAAGCGCAGCTCGGCGGCGATCTATGCGAACCGTTGCCCGGCGG

4166R AAGCCCAACGCGCTCCGAAGCGCAGCTCGGCGGCGATCTGTGCGAACCGTTGCCCGGCGG

1501R AAGCCCAACGCGCTCCGAAGCGCAGCTCGGCGGCGATCTGTGCGAACCGTTGCCCGGCGG

***************************************.********************

M1SNM1R CCGTGGGCGGAGTAGCGTGATCGCGAACCGAGGAAGGAGATTCGCC

FN13R CCGTGGGCGGAGTAGCGTGATCGCGAACCGAGGAAGGAGATTCGCC

4166R CCGTGGGCGGAGTAGCGTGATCGCGAACCGAGGAAGGAGATTCGCC

1501R CCGTGGGCGGAGTAGCGTGATCGCGAACCGAGGAAGGAGATTCGCC

**********************************************

Alignment of *P. stutzeri* A1501 IRRight (1501R) and *P. stutzeri* KOS6 IRRight (KOS6R). Asterisks indicate nucleotide identities.

KOS6R CCTTGCGTCGGGCTTCGCTG--TGCTCAGTGAAGCCCAAC----------TCAAATGAAG

1501R CCATGCGTTGAGCTTCGCTGCACGCCCGGCCCCGACCTACGCCTCGTAATCCGTAGGTTG

**:***** *.********* ** *.* ..*.**:** *.:* *::*

KOS6R TGTT------------------GCCCAACGTTTCACGGACTCGCAGCAG-----------

1501R GGTTGATACGCGCAGCATCGAAGCCCAACGCG-CTCCGAAGCGCAGCTCGGCGGCGATCT

*** ******** *:* **. ******:

KOS6R -------TCGTTGCCCGGCGCTCGCTGGCGGAGTAGCGTGACTGCAAACCGAGGACGGAG

1501R GTGCGAACCGTTGCCCGGCGGCCGTGGGCGGAGTAGCGTGATCGCGAACCGAGGAAGGAG

************ ** *************** **.*********.****

KOS6R AATCGAC

1501R ATTCGCC

*:***.*

Alignment of *P. stutzeri* A1501 IRRight (1501R) and *P. azotifigens* 17556 IRRight (17556R). Asterisks indicate nucleotide identities.

17556R GCTCGCCGGGCCTTCGCTGCGCCGAATCCTGGCGACCCAGCCCAACCTGCGTTCCCCGGT

4166R -----CCATGCGTTG---------------AGCTTCGCTGCACGCCCG----GCCCCG--

**. ** ** .** :* *:**.*..** *****

14556R GTAACCTGGACAAGCTCGCCCCGAGATCGGGCCTCCCACAAAAGCGGCGTCGCCCACCGA

4166R ---ACCT--------ACGCCTCGTAATCCG------------------------------

**** :**** **:.*** *

14556R GTCCGGTGGGAGGCACCCTCGCGGCGAACGCGGGCGCGTAGAAGCCCAACGTTTTTCCTA

4166R --TAGGTTGGGTTGATACGCGCAGC------------ATCGAAGCCCAACG-------CG

*** **. * .* ***.** .*.*********** .

14556R CGCCGAGGCTCGAGTTCAACAGCGATCCGCCCGAATCGTTGCCCGGCGGGCATGGGCGGA

4166R CTCCGAAGCGC-AGCTCGGCGGCGATCTGTGCGAACCGTTGCCCGGCGGCCGTGGGCGGA

* ****.** * ** **..*.****** * **** ************* *.********

14556R GTAGCGTGGCTGTGAAACCGACAATGGAGATTCGCC

4166R GTAGCGTGATCGCG-AACCGAGGAAGGAGATTCGCC

********. * * ****** .*:***********

Alignment of *P. stutzeri* Gr65 IRRight (Gr65R) and *P.stutzeri* DSM 4166 IRRight (4166R). Asterisks indicate nucleotide identities.

4166R CCATGCGTTGAGCTTCGCTGCACGCC-------CGGCCCCGAC-----------------

Gr65R CCATGCGTTGGGCTTCGCTACGCTCAACCCAGGCGGCCCCGCCGAACCAACGATGTCGGC

**********.********.*.* *. ********.*

4166R ----------------------------------------CTACGCCTCGTAATCCG---

Gr65R GGGGATGGTAGGTTGGATCGGACGGCGTTCCGCTGAAACGCGTAGCGTCGAAGCCCAGCT

* :.** ***:*. **.

4166R ------------------------------------------------------------

Gr65R CACACGAAGGTAACGCTTGGCACCCGTCCCGTTGGGGGCTTGGCTTGGTTGTGACCGTGC

4166R ----------------------------------------------------------TA

Gr65R GTTGGGCTTGGCTGTGCCCAGGCTGCGCGCCCCGACTCAACAGTTGGATGTCGACGGGGT

:

4166R GGTTGGGTTGATACGCGCAGCATCGAAGCCCAACGCGCTCCGAAGCGCAGCTCGGCGGCG

Gr65R GATCGGGTCGAGACGCGCAGCGTCGAAGCCCAACTCACATGAAGGCGATGCCCAACGTTT

*.* **** ** *********.************ *.*: .*.***.:** *..**

4166R ATCTG-----TGCGAACCGTTGCCCGGCGGCCGTGGGCGGAGTAGCGTGATCGCGAACCG

Gr65R CACGGGCTCGCATGAGCCGTTGCCCGGCGACCGTGGGCGGAGTAGCGTGACTGCAAACCG

.:* * . **.*************.******************** **.*****

4166R AGGAAGGAGATTCGCC

Gr65R ACGATGGAGACTCGCC

* **:***** *****
