# Supplementary material for: The Nitrogen-Fixation Island Insertion Site Is Conserved in Diazotrophic Pseudomonas stutzeri and Pseudomonas sp. Isolated from Distal and Close Geographical Regions
Source: PLoS One. 2014 Sep 24;9(9):e105837. doi: 10.1371/journal.pone.0105837 (PMC4174501; doi:10.1371/journal.pone.0105837)
Supplement: File S3 — Nucleotide sequences of P. stutzeri KOS6 IRLeft and IRRight. (DOCX) [file pone.0105837.s004.docx]

**File S3. Nucleotide sequence *P. stutzeri* KOS6 IRLeft and IRRight**

Nucleotide sequence *P. stutzeri* KOS6 IRLeft and IRRight, KOS6L and KOS6R, respectively. The direct repeats identified in KOS6L are underlined. The inverted repeats identified in KOS6R are denoted by bold letters.

>KOS6L

GTTGTGGAGCAGGCCCCCGCCTGCACAGCGGAAATGCATGCTCCAGACCCAAAAGCATCGCCCCGAGGTCGGGCCTCCCACAAAGGCAGCGAGTGCACACCATGCCCGGTGGGAGGCGCGCCCTCGCGGCGAAGCAGGCTGAGGCCTGCCGGAAACTCACCGGCAAGCCATCGATCTTTCCGGTGCCGCCATGCATCTGGTCGCGGCCTGACATTAGGCTGCTATTGCGCAGCGGAAATGCACGCTCCAGACCCAAAAGCATCGCCCCGAGGTCGGGCCTCCCGCAAAGGCAGCGAGCGCACACAGGCCTCGTGGGAGGCGCGACCTCGCGGCGAAGCAGGCTAAGGCCTGCCGGAAGCTCACCGGCAGCCATCGATCTTTCCGGTGCCGCCATCTGGTCGCGGCCTGACATTAGGCTGCTATTGCGCAGCGGGAATGCACGCTCCAGGCCCAAAAGCATGGCCCCGAGGTCGGGCCTCCCGCAAAGGCAGCGAGCGCACACAGGCCTCGTCGTGGGAGGCGCGCCCTCGCGGCGAAGCAGGCCGCAGGCCTGCCGGAAGAATCTGCATGCGC

>KOS6R

CCTTGCGTCGGGCTTCGCTGTGCTCAGTGAAGCCCAACTCAAATGAAGTGTTGCCCAACGTTTCACGGACTCGCAGCAGTCGTTGCCCG**GCGCTCGCTG**GCGGAGTAGCGTGACTGCAAACCGAGGACGGAGAATCGAC
