# Supplementary material for: The Nitrogen-Fixation Island Insertion Site Is Conserved in Diazotrophic Pseudomonas stutzeri and Pseudomonas sp. Isolated from Distal and Close Geographical Regions
Source: PLoS One. 2014 Sep 24;9(9):e105837. doi: 10.1371/journal.pone.0105837 (PMC4174501; doi:10.1371/journal.pone.0105837)
Supplement: File S4 — Nucleotide sequences of P. azotifigens DSM 17556 IRLeft and IRRight. (DOCX) [file pone.0105837.s005.docx]

**File S4. Nucleotide sequences of *Pseudomonas azotifigens* DSM 17556 IRLeft and IRRight**

Nucleotide sequences of *Pseudomonas azotifigens* DSM 17556 IRLeft and IRRight, 17556L and 17556R respectively. The identical direct repeats identified in are denoted by different coloured letters.

>17556L

CGTGACGTGAGCCGGCGGTTGCTGCCGCTCTGGCTGGCCGCGGGTGCGTCGATGCTGGCCCTGGCCTGGTGGGGCTGGGCGCAGGGCGGGATGGCGCTGCTGCAGCTGGGCGTCGGAGTCTGCTGAGCGGGGCGCGGGCGTCGGGCTTTTCGTGAGCGGTCTCGACCCGTGAACTCAAGGGGCCAGGTTCGCGGTCGAGACCGCTCCCACAAAAGCATCGCCCCGAGGGCGGGCCTCCCACGAAAGGCGATTCTGTGGCAGGCGCGCCCTCGCGGCGATGCAGGCCGCAGGCCTGCCAGAATCTCATCGGTCTTTCAACGCCCCGGAGATCATCTTCGCCTCGGTACGATCAGCCTACGCTCGCCCCTG

>17556R

GCTCGCCGGGCCTTCGCTGCGCCGAATCCTGGCGACCCAGCCCAACCTGCGTTCCCCGGTGTAACCTGGACAAGCTCGCCCCGAGATCGGGCCTCCCACAAAAGCGGCGTCGCCCACCGAGTCCGGTGGGAGGCACCCTCGCGGCGAACGCGGGCGCGTAGAAGCCCAACGTTTTTCCTACGCCGAGGCTCGAGTTCAACAGCGATCCGCCCGAATCGTTGCCCGGCGGGCATGGGCGGAGTAGCGTGGCTGTGAAACCGACAATGGAGATTCGCC
