# Supplementary material for: The Nitrogen-Fixation Island Insertion Site Is Conserved in Diazotrophic Pseudomonas stutzeri and Pseudomonas sp. Isolated from Distal and Close Geographical Regions
Source: PLoS One. 2014 Sep 24;9(9):e105837. doi: 10.1371/journal.pone.0105837 (PMC4174501; doi:10.1371/journal.pone.0105837)
Supplement: File S5 — Phylogenetic trees based on ITS1 (A), nifD (B), narJ (C), napA (D), nirS (E), nosZ (F), nasA (G) and nasB (H) showing the relationships among the Pseudomonas strains and references strains. (DOCX) [file pone.0105837.s006.docx]

**File S5**. **Phylogenetic trees based on ITS1 (A), *nifD* (B), *narJ* (C), *napA* (D), *nirS* (E), *nosZ* (F), *nasA* (G) and *nasB* (H) showing the relationships among the *Pseudomonas* strains and references strains.** The trees were constructed by the neighbour-joining method using MEGA v.5 [31]. Reference strains are highlighted in bold and type strains with superscript T. Numbers at nodes indicate levels of bootstrap support (using 1000 replicates). Scale bars indicate the number of substitutions per site of the genes. The accession numbers for the sequences of the reference strains are indicated within parentheses.

**S5. A**

**S5. B**

**S5. C**

**S5. D**

**S5. E**

**S5. F**

**S5. G**

**S5. H**
