# Supplementary material for: The Nitrogen-Fixation Island Insertion Site Is Conserved in Diazotrophic Pseudomonas stutzeri and Pseudomonas sp. Isolated from Distal and Close Geographical Regions
Source: PLoS One. 2014 Sep 24;9(9):e105837. doi: 10.1371/journal.pone.0105837 (PMC4174501; doi:10.1371/journal.pone.0105837)
Supplement: Table S1 — Beneficial properties of Pseudomonas strains used in this study. (DOCX) [file pone.0105837.s007.docx]

**Table S1**. Beneficial properties of *Pseudomonas* strains used in this study

| A/A | Strain | Location | Source | Plant variety | nmol eth h−1 mg−1 protein | Reference |
| --- | --- | --- | --- | --- | --- | --- |
| 1 | **Gr16** | Skourta, Viotia | *Triticum durum* | capeiti | 9.3±2.9 ab | Venieraki et al., 2011a |
| 2 | **Gr 17** | Skourta, Viotia | *Triticum durum* | capeiti | 6.6±3.1 ab | Venieraki et al., 2011a |
| 3 | **Gr 18** | Skourta, Viotia | *Triticum durum* | capeiti | 30.1±2.3 c | Venieraki et al., 2011a |
| 4 | **Gr 19** | Skourta, Viotia | *Triticum durum* | capeiti | 11.4±3.5 b | Venieraki et al., 2011a |
| 5 | **Gr 20** | Skourta, Viotia | *Triticum durum* | capeiti | 29.8±0.3 c | Venieraki et al., 2011a |
| 6 | **Gr 21** | Skourta, Viotia | *Triticum durum* | capeiti | 10.2±3.1ab | Venieraki et al., 2001a |
| 7 | **Gr 45** | Thessaloniki, Macedonia | *Triticum aestivum* | Generoso | 6.8+1.6 a | This study |
| 8 | **Gr 46** | Thessaloniki, Macedonia | *Triticum aestivum* | Generoso | 7.5+1,3 a | Venieraki et al., 2011b |
| 9 | **Gr 50** | Thessaloniki, Macedonia | *Hordeum vulgare* | Carina | 4.5+0.2 a | Venieraki et al., 2011b |
| 10 | **Gr 57** | Kileler, Thessaly | *Hordeum vulgare* | local | 11.8±2.5 b | This study |
| 11 | **Gr 65** | Kileler, Thessaly | *Hordeum vulgare* | local | 13.4±3.3 b | This study |

Results are means ± SD of three experiments conducted separately under identical conditions. Number in a column followed by the same letter is not significantly different at the 0.05 level using Duncan’s multiple-range test. Nitrogenase activity was measured after growth in NFb semisolid medium.
