# Supplementary material for: The Nitrogen-Fixation Island Insertion Site Is Conserved in Diazotrophic Pseudomonas stutzeri and Pseudomonas sp. Isolated from Distal and Close Geographical Regions
Source: PLoS One. 2014 Sep 24;9(9):e105837. doi: 10.1371/journal.pone.0105837 (PMC4174501; doi:10.1371/journal.pone.0105837)
Supplement: Table S2 — List of primers used and PCR cycling conditions. (DOCX) [file pone.0105837.s008.docx]

**Table S2**. List of primers used and PCR cycling conditions

| Primer | Sequence 5'-3' | Conditions | Reference |
| --- | --- | --- | --- |
| fD1  rD1 | AGA GTT TGA TCC TGG CTC AG  AAG GAG GTG ATC CAG CC | 4’94ºC, 34x(1’94ºC, 1’48ºC, 4’72ºC), 10`72 ºC | Weisburg et al., 1991 |
| A1F (Rrn16S )  A2R (Rrn23S ) | 5’ GAAGTCGTAACAAGG  CAAGGCATCCACCGT | 4’94ºC, 34x(1’94ºC, 1’55ºC, 4’72ºC), 10`72 ºC | Lin & Stewart, 1998 |
| *nifD*-2f  *nifD*-1r | CATCGGIGACTCAAYATYGGYGG  CCCAIGARTGCATYTGICGGGAA | 4’94ºC, 34x(1’94ºC, 1’52ºC, 4’72ºC), 10`72 ºC | Fedorof et al., 2008 |
| *nirS*-F1  *nirS*-R1 | CACGGYGTBCTGCGCAAGGGCGC  CGCCACGCGCGGYTCSGGGTGGTA | 4’94ºC, 34x(1’94ºC, 1’52ºC, 4’72ºC), 10`72 ºC | This study |
| *nosZ* F  *nosZ* R | CGYTGTTCMTCGACAGCCAG  CATGTGCAGNGCRTGGCAGAA | 4’94ºC, 34x(1’94ºC, 1’52ºC, 4’72ºC), 10`72 ºC | This study |
| *napA* PST_1268 F  *napA* PST_1268 R | CCTTCTCCACCCACATGGCGCTGGGC  CCAGCCSTCSGCCTGCGGCACSGC | 4’94ºC, 34x(1’94ºC, 1’63ºC, 4’72ºC), 10`72 ºC | This study |
| *narJ*-533-F  *narJ*-533-F | CCGAAGGCGACCTGATGGAC  CACCGGGCTTTCCTCGCGAG | 4’94ºC, 34x(1’94ºC, 1’52ºC, 4’72ºC), 10`72 ºC | This study |
| *nasA* PST_2411 F  *nasA* PST_2411 R | CTGTGCATGTCYTCGGCGGT  GCCAGGTGCAGGTTGATCAG | 4’94ºC, 34x(1’94ºC, 1’56ºC, 4’72ºC), 10`72 ºC | This study |
| *nasB* PST_2409 F  *nasB* PST_2409 R | CCTGCTCGGSCTVGAGGCGGC  CTCTTCGGCTTGACGTCGGCCG | 4’94ºC, 34x(1’94ºC, 1’60ºC, 4’72ºC), 10`72 ºC | This study |
| REP 1R-I  REP 2-I | IIIICGICGICATCIGGC  ICGICTTATCIGGCCTAC | 7’95ºC, 30x(30’’90ºC, 1’, 40ºC, 8’65ºC), 16`65 ºC | Versalovic et al., 1991 |
| IRLeft F  IRLeft R | GGCAAGTTCGTCGGSCTGGCGCTGG  CGTACTTGTAGAAGTTCCAGCGCGGCG | 4’94ºC, 34x(1’94ºC, 1’62ºC, 4’72ºC), 10`72 ºC | This study |
| IRRight_F  IRRight_R | GTGATGCGCCTGCTGCTGGC  GCTGCGCGGGTCCTTCATGATC | 4’94ºC, 34x(1’94ºC, 1 55ºC, 4’72ºC), 10`72 ºC | This study |
| PST1301_F  PST1304_R | GATCATGAAGGACCCGCGCAGCGGCC  CTACAAACAACTATGCGAACAGGACG | 30’’98ºC, 34x(10’’98ºC, 30’’, 69ºC, 4’72ºC), 2`72 ºC | This study |
| PST1357­_F  PST1360_F | CCGCATCAACCTGATCGCCGAGC  GTCTGGGTCATGGTGAAGGTCAC | 30’’98ºC, 34x(10’’98ºC, 30’’, 69ºC, 4’72ºC), 2`72 ºC | This study |

Fedorof DN, Ivanova EG, Doromina NV, Yu A, Trotsenko A (2008) A new system of degenerate oligonucleotides primers for detection and amplification of *nifHD* genes. Mikrobiologiya 77:286-288

Versalovic J, Koeuth T, Lupski JR (1991) Distribution of repetitive DNA sequences in Eubacteria and application to fingerprinting of bacterial genomes. Nucleic Acids Res 19:6823–6831

Weisburg WG, Barns SM, Pelletier DA, Lane DJ (1991) 16S ribosomal DNA amplification for phylogenetic study. J Bacteriol 17: 697–703
